# Analyses of the genetic diversity and population structures of *Histoplasma capsulatum* clinical isolates from Mexico, Guatemala, Colombia and Argentina, using a randomly amplified polymorphic DNA-PCR assay

**DOI:** 10.1017/S0950268819000931

**Published:** 2019-05-29

**Authors:** J. H. Sahaza, E. Duarte-Escalante, C. Canteros, G. Rodríguez-Arellanes, M. R. Reyes-Montes, M. L. Taylor

**Affiliations:** 1 Unidad de Micología Médica y Experimental, Corporación para Investigaciones Biológicas, Medellín, Colombia; 2 Departamento de Microbiología-Parasitología, Unidad de Micología, Facultad de Medicina, Universidad Nacional Autónoma de México (UNAM), Mexico City, Mexico; 3 Departamento de Micología, Instituto Nacional de Enfermedades Infecciosas ‘Dr Carlos G. Malbrán’, Buenos Aires, Argentina

**Keywords:** Clonal population, genetic diversity, *Histoplasma capsulatum*, Latin American isolates, recombinant population

## Abstract

We studied the genetic diversity and the population structure of human isolates of *Histoplasma capsulatum*, the causative agent of histoplasmosis, using a randomly amplified polymorphic DNA-polymerase chain reaction (RAPD-PCR) assay to identify associations with the geographic distribution of isolates from Mexico, Guatemala, Colombia and Argentina. The RAPD-PCR pattern analyses revealed the genetic diversity by estimating the percentage of polymorphic loci, effective number of alleles, Shannon's index and heterozygosity. Population structure was identified by the index of association (*I*_A_) test. Thirty-seven isolates were studied and clustered into three groups by the unweighted pair-group method with arithmetic mean (UPGMA). Group I contained five subgroups based on geographic origin. The consistency of the UPGMA dendrogram was estimated by the cophenetic correlation coefficient (*CCCr* = 0.94, *P* = 0.001). Isolates from Mexico and Colombia presented higher genetic diversity than isolates from Argentina. Isolates from Guatemala grouped together with the reference strains from the United States of America and Panama. The *I*_A_ values suggest the presence of a clonal population structure in the Argentinian *H. capsulatum* isolates and also validate the presence of recombining populations in the Colombian and Mexican isolates. These data contribute to the knowledge on the molecular epidemiology of histoplasmosis in Latin America.

## Introduction

*Histoplasma capsulatum*, the causative agent of the systemic mycosis histoplasmosis, is a dimorphic ascomycete that grows at 25 °C in the mycelial phase in nature (saprobe and infective morphotype) and at 37 °C in the yeast phase, when it infects susceptible mammalian hosts (parasitic and virulent morphotype). The morphotype transition of *H. capsulatum*, from mycelial to yeast phase, is a necessary step for establishing histoplasmosis infection and the successive clinical manifestations of histoplasmosis disease. Therefore, the yeast morphotype promotes the expression of genes that are associated with the production of virulence factors required for fungal pathogenicity.

Several molecular assays have been developed to genotype *H. capsulatum*, contributing to a better understanding of the epidemiology and genetic diversity of this fungus [1–11]. These findings have revealed that far from being a single and well-resolved species, *H. capsulatum* consists of a number of genetically distinct groups of isolates that frequently correlate with a precise geographic origin. Currently, *H. capsulatum* is considered to be a complex of cryptic species according to the phylogenetic species concept [1, 2].

Based on analyses of 137 *H. capsulatum* isolates from 25 countries, seven phylogenetic species belonging to the *H. capsulatum* complex were identified: North American class 1 (NAm 1), North American class 2 (NAm 2), Latin American group A (LAm A), Latin American group B (LAm B), Australian, Netherlands and African [2]. Teixeira *et al*. [3] proposed that *H. capsulatum* has at least 11 cryptic phylogenetic species, six of which have been reported as new species (RJ, LAm A1, LAm A2, LAm B1, LAm B2 and BAC1). Recently, Sepúlveda *et al*. [4], using whole-genome sequences, reported new data describing the phylogeny of *H. capsulatum* through the analysis of 30 isolates representing areas where histoplasmosis is recognised to be endemic. These researchers proposed a rearrangement of the *Histoplasma* genus by renaming four species that were genetically isolated: lineage H81 from Panama as *H. capsulatum sensu stricto* Darling 1906; phylogenetic species NAm 1 as *H. mississippiense* sp. nov.; phylogenetic species NAm 2 as *H. ohiense* sp. nov. and phylogenetic species LAm A as *H. suramericanum* sp. nov.

The predominance of a particular population structure of *H. capsulatum* in different geographic regions could explain the high and low genetic diversity of this pathogen and suggests that distinct populations of this fungus have been spread in the environment, according to its sexual (recombining population) or asexual (clonal population) reproduction. Recombining populations of *H. capsulatum* were detected in the South American clade (SAm *Hcc*), which showed a large association index value, suggesting sexual recombination [1]. Carter *et al*. [5] reported a recombining population structure in *H. capsulatum* strains that were isolated in Indianapolis, United States of America, suggesting that *H. capsulatum* was able to recombine in nature. Carter *et al*. [6] also identified important genetic differences between strains of *H. capsulatum* from North (Indianapolis) and South America (Colombia), through the analyses of *H. capsulatum* population structures, using single nucleotide polymorphisms and microsatellite markers. Overall, Kasuga *et al*. [1, 2] suggested that NAm fungal populations are less diversified than LAm populations and that they are probably associated with a more recent expansion of the fungus within the North American region. Furthermore, considering genetic population structure analyses of the LAm and NAm clades, Teixeira *et al*. [3] have suggested that there are multiple phylogenetic species within the LAm A population in the Latin American region and they have proposed the existence of three main monophyletic species within the NAm population (NAm 1, NAm cat-associated and NAm 2) in the USA.

The randomly amplified polymorphic DNA using the polymerase chain reaction (RAPD-PCR) method has been applied for typifying genetic diversity and to identify *H. capsulatum* isolates, regarding their DNA polymorphisms and their distinct geographic origins [12–15]. Boldo *et al*. [16] analysed genetic distances, genetic similitude, gene flow and population structures of clinical and reference strains of *Candida glabrata* from several geographic areas by RAPD-PCR, providing support to the efficacy of this method for studying many genetic parameters.

The aim of this work was to add more data on *H. capsulatum* genetic diversity, in relation to the population structure of several human clinical fungal isolates from the Latin American region. In an effort to identify any possible association between fungal genetic diversity and population structure, considering the geographic origin of the studied isolates, we used the RAPD-PCR assay as it was successfully employed before to generate data regarding the population structure of other fungal species.

## Methods

### Histoplasma capsulatum

We studied 34 human clinical isolates: 13 from Mexico, eight from Argentina, 11 from Colombia and two from Guatemala. Three clinical strains from the American Type Culture Collection (ATCC) were used as references (G-217B ATCC 26032, North American class 2 (NAm 2 clade) and Downs ATCC-38904, North American class 1 clade (NAm 1), both of which are from the USA, as well as lineage G-186B ATCC-26030 from Panama) (see Table 1). Most of the fungal samples came from the *Histoplasma capsulatum* Culture Collection of the Laboratorio de Inmunología de Hongos (LIH) of the Departamento de Microbiología-Parasitología, Facultad de Medicina, UNAM, Mexico (http://www.histoplas-mex.unam.mx), which is registered in the database of the World Data Centre for Microorganisms (WDCM) of the World Federation for Culture Collections under the acronym and number LIH-UNAM WDCM817. Isolates were preserved in Sabouraud-agar supplemented with sterile mineral oil at 4 °C. The isolates were routinely cultured in brain heart infusion-agar (Bioxon, Becton-Dickinson, Mexico City, CDMX, MX) at 25–28 °C under laboratory conditions.

**Table 1.** Clinical isolates of *H. capsulatum*

Acronym	Clade/phylogenetic species/lineage	Geographic origin	Clinical form	Immune-compromise
EH-316	LAm A^a^	MX	D	HIV+
EH-317	LAm A^a^	MX	D	HIV+
EH-319	LAm A^a^	MX	D	HIV+
EH-323	LAm A^b^	MX	D	HIV+
EH-324	LAm A^b^	MX	D	HIV+
EH-325	LAm A^a^	MX	D	HIV+
EH-326	LAm A^b^	MX	D	HIV+
EH-327	LAm A^b^	MX	D	HIV+
EH-328	LAm A^b^	MX	D	HIV+
EH-355	LAm A^b^	MX	ND	HIV+
EH-356	LAm A^b^	MX	ND	HIV+
EH-357	LAm A^b^	MX	D	HIV+
EH-359	LAm A^a^	MX	MC	ND
H.1.12.96	LAm A^a^	GT	MC	ND
H.1.02.W	LAm A^a^	GT	P	ND
92590	LAm B^b^	AR	MC	HIV+
951814	LAm B^b^	AR	MC	HIV+
993444	LAm B^b^	AR	MC	HIV+
993445	LAm B^b^	AR	MC	HIV+
993446	LAm B^b^	AR	MC	HIV+
993267	LAm B^b^	AR	MC	HIV+
01558	LAm B^b^	AR	MC	HIV+
01739	LAm B^b^	AR	MC	HIV+
LA	LAm A^b^	CO	D	HIV+
WCh	LAm A^b^	CO	MC	HIV+
GLI	LAm A^b^	CO	MC	HIV+
DS	LAm A^b^	CO	D	ND
AP	LAm A^b^	CO	D	ND
LF	LAm A^b^	CO	D	ND
RG	LAm A^b^	CO	P	HIV+
WE	ND	CO	P	HIV+
JG	LAm B^b^	CO	MC	HIV+
MZ2	LAm A^b^	CO	MC	SLE
GeM	LAm A^b^	CO	ND	ND
G-186B	Lineage^a^	PA	P	ND
G-217B	NAm 2^a^	USA	ND	ND
Downs	NAm 1^a^	USA	MC	Diabetes

MX, Mexico; GT, Guatemala; AR, Argentina; CO, Colombia; PA, Panama; USA, United States of America; P, pulmonary; D, disseminated; MC, mucocutaneous; ND, not determined; HIV, human immunodeficiency virus; SLE, systemic lupus erythematosus.

a Phylogenetic species registered by Kasuga *et al*. [2].

b M.L. Taylor, unpublished data.

### Fungal DNA

*H. capsulatum* whole-cell DNA was obtained from mycelial-phase cultures as described by Reyes-Montes *et al*. [14]. The concentration of each DNA sample was quantified in an Epoch microplate spectrophotometer (BioTek Instruments Inc., Winooski, VT, USA) at 260–280 nm and checked against standard lambda phage DNA concentrations on 0.8% (w/v) agarose gel electrophoresis. Finally, DNA samples were frozen at −20 °C until required.

### RAPD-PCR assay

The arbitrary primers used were: 1253 (5′-GTTTCCGCCC-3′), 1281 (5′-AACGCGCAAC-3′) and 1283 (5′-GCGATCCCCA-3′) (Sigma-Aldrich, St. Louis, MO, USA), as previously reported [12, 13]. RAPD-PCR was performed using one-primer [12, 13] and two-primer methods [17]. The assays were performed in a 25 µl reaction, using 10 ng of *H. capsulatum* DNA and containing 10 mM Tris-HCl pH 8.3, 2.5 mM MgCl_2_, 200 µM of each dNTP, 100 pmol of each primer and 1 U of *Taq* DNA polymerase (Perkin Elmer Cetus, Wellesley, MA, USA). PCR amplification was conducted in a thermal cycler (Perkin Elmer Cetus), programmed as follows: one cycle at 94 °C for 5 min, followed by 45 cycles of 30 s at 92 °C, 1 min at 35 °C and 1 min at 72 °C, for DNA denaturing, annealing and extension, respectively. Amplifications were performed twice for two independent assays, and all of the assays were conducted in the same laboratory, using the same enzyme, reagent brands and thermocycler.

The RAPD-PCR patterns were photographed and analysed according to digital images of GelRed-stained agarose plates (1.5% w/v), containing a 123-bp DNA ladder (Gibco BRL, Life Technologies, Grand Island, NY, USA) as the molecular size standard. The photographed amplified fragment patterns were processed with an image analysis system (Adobe^®^ Photoshop^®^, Adobe Systems Inc., USA) and manually coded and translated into binary data that indicated either their presence (1) or absence (0).

A pairwise similarity matrix was developed, and cluster analysis was performed using the unweighted pair group method with arithmetic averages (UPGMA). The distortion of the inferred tree was estimated by means of the cophenetic correlation coefficient (*CCCr*) [18] with the nonparametric Mantel test [19] and the bootstrap (bt) method [20]. Multivariate statistical methods were performed with the NTSYS-PC program version 2.0 [18], TreeView version 1.6.6 (http://taxonomy.zoology.gla.ac.uk./rod/rod/html), and FreeTree version 0.9.1.50 [21].

### Genetic diversity estimation

Genetic diversity data, such as the percentage of polymorphic loci (*P*), effective number of alleles (*n*_e_) and Shannon's index (*I*), were calculated by assuming that each phenotypic marker represented a distinct locus [22], whereas Nei's genetic diversity or expected heterozygosity (*h*) was determined using allelic frequencies [23], according to Zhivotovsky's Bayesian method [24]. All of these estimation data were calculated using the POPGENE program, version 1.31 [25].

### Population structure determination

To distinguish between clonal and recombinant structures, we used the index of association (*I*_A_) [26], a statistical test that measures the degree of non-random association between alleles at different loci (linkage disequilibrium), implemented in LIAN version 3.5 [27].

## Results

### RAPD-PCR analyses

**Fig. 1.**
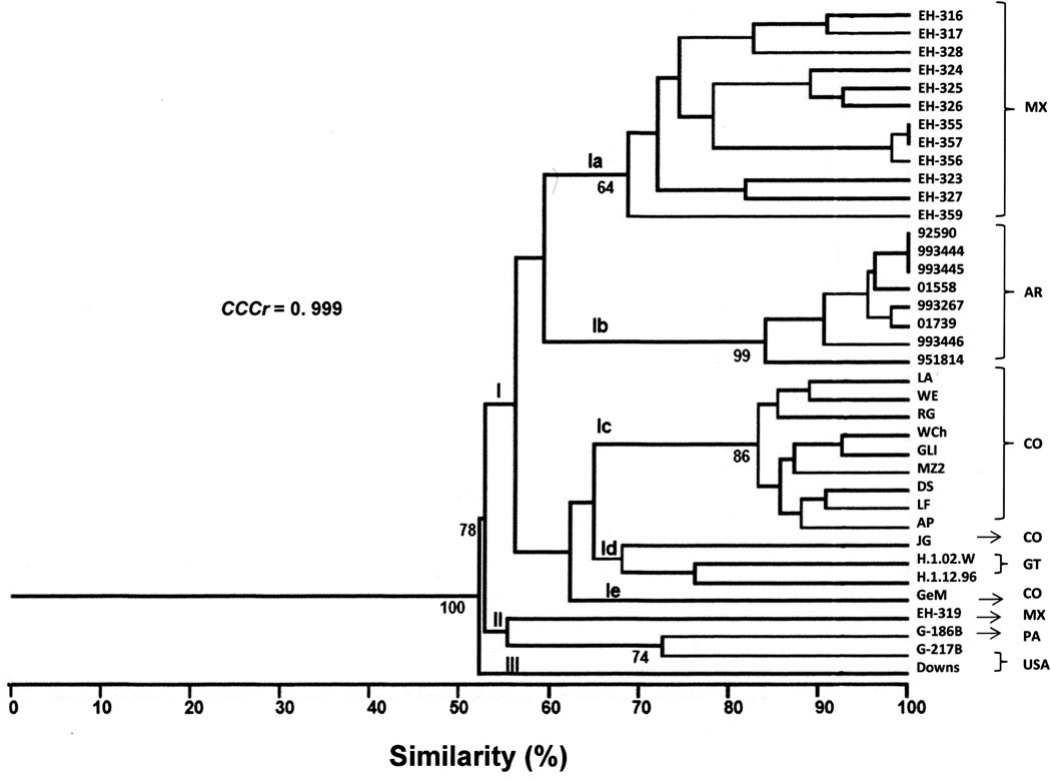
Percentages of similarity between *H. capsulatum* clinical isolates and the reference strains studied. The dendrogram was constructed by using the UPGMA method, analysing the generated RAPD-PCR patterns. Bt values higher than 60% are represented in this figure to support the clustering. The value of the *CCCr* is depicted in this figure along with a significant *P* = 0.001. MX, Mexico; AR, Argentina; CO, Colombia; GT, Guatemala; PA, Panama; USA, United States of America.

A total of 58 RAPD markers were determined from the one- and two-primer RAPD-PCR patterns (data not shown). The dendrogram generated with 34 *H. capsulatum* isolates from Latin American countries and the reference strains studied yielded three groups (Fig. 1). Group I had a 100% bt value and included five subgroups. Subgroup Ia included most of the isolates from Mexico, with 70% similarity among them; however, isolate EH-319 clustered with the reference strains from Panama (G-186B) and the USA (G-217B). Subgroup Ib included all of the isolates from Argentina, which showed the highest similarity (84%) in this study. Most of the isolates from Colombia were found in subgroup Ic (83%), with the exceptions of isolate JG that was placed in subgroup Id together with isolates H.1.02.W and H.1.12.96 from Guatemala (these three isolates clustered with a similarity of 64%), and isolate GeM that was the lone member of subgroup Ie. Group II included one isolate from Mexico (EH-319) and the reference strains from Panama (G-186B) and the USA (G-217B), all of which clustered with a similarity of 55%. Finally, Group III included only the reference strain (Downs) from the USA.

The *CCCr* = 0.999 (*P* = 0.0004) suggested that the dendrogram accurately represented the original genetic similarities between *H. capsulatum* isolates and the reference strains studied (Fig. 1). These results highlight that subgroup Ib was the most consistent, because all eight Argentinian isolates were clustered together, and six of them (92590, 993444, 993445, 01558, 993267 and 01739) were related with a 92% similarity (Fig. 1).

### Genetic diversity

The genetic parameters of *H. capsulatum* isolates and the reference strains revealed that most genetic diversity was found in the Mexican fungal population, followed by the Colombian and Argentinian populations. Thus, the highest values of the polymorphic loci, effective number of alleles, Shannon's index and heterozygosity corresponded to the Mexican isolates (Table 2). In contrast, the lowest values for genetic diversity parameters were associated with the Argentinian isolates (see Table 2).

**Table 2.** Parameters of the genetic diversity of the *H. capsulatum* populations studied

Population	*P* (%)	*n*_e_ ± s.e.	*I* ± s.e.	*h* ± s.e.
MX	87.27	1.436 ± 0.096	0.400 ± 0.067	0.261 ± 0.049
CO	63.64	1.398 ± 0.033	0.334 ± 0.088	0.225 ± 0.063
AR	25.45	1.153 ± 0.109	0.131 ± 0.086	0.088 ± 0.060

*P*, polymorphic loci; *n*_e_, effective number of alleles; *I*, Shannon's index; *h*, heterozygosity; s.e., standard error. MX, Mexico; CO, Colombia; AR, Argentina.

### Population structure

The association index values estimated from all the studied isolates, indicated that *H. capsulatum* had a recombinant population structure in the Mexican (*I*_A_ = 0.2634) and Colombian (*I*_A_ = 0.3627) populations, whereas the Argentinian population exhibited a clonal structure according to its *I*_A_ value (0.7324).

## Discussion

In the current study, we demonstrated that RAPD-PCR is an adequate method to generate valuable information concerning the genetic diversity and the population structure of the *H. capsulatum* clinical isolates here analysed, even though this molecular tool has been erroneously criticised in the past for non-reproducible results [28]. However, the robustness of this method is highlighted by its effectiveness and simple development, under well-controlled experimental conditions. This method can be useful for identifying slight differences between closely related groups of fungal isolates, because it is reproducible under standardised conditions [12–14].

The genetic diversity of *H. capsulatum* isolates, as determined by UPGMA analysis of RAPD-PCR patterns, revealed that fungal isolates from Mexico, Argentina, Colombia and Guatemala share genetic similarities that justified their clustering into only one group (group I), which was supported by a high bt value. In this group, five geographically defined subgroups were distinguished, suggesting the separation of the isolates according to their geographic origin, which was probably associated with genetic changes within the same subgroup or geographic population. The latter is in agreement with the hypothesis that *H. capsulatum* radiation started its earliest evolution in the Latin American region millions of years ago, as has it been proposed by Kasuga *et al*. [2]. This hypothesis may also be related to certain behaviours of the fungus, such as its association with some mammalian hosts, including bats, which were able to spread the fungus over large geographic distances [3, 7]. Although there is a close relationship between isolates and their geographic distribution, genetic diversity analyses of RAPD-PCR patterns, through parameters such as *P*, *n*_e_, *I* and *h* values, provide robust data indicating a high level of diversity within *H. capsulatum* isolates from Mexico and Colombia, a feature that was corroborated with their *I*_A_ values calculated, which suggest recombining population structure in these isolates.

High levels of genetic diversity in *H. capsulatum* could be the result of some processes such as hybridisation or introgression, incomplete lineage sorting and recombination. Teixeira *et al*. [3] suggested that an *H. capsulatum* strain (EH-315), isolated from an infected bat, and belonging to a new phylogenetic species named BAC1, could be the result of an introgression process between two different clusters of *H. capsulatum* that generated a new genotype. Sepúlveda *et al*. [4] have suggested that introgression, rather than incomplete lineage sorting, contributes to the genetic diversity of *Histoplasma*, since the species of this fungus could exchange genetic material through this process. More recently, introgression has been proposed as a possible process for generating new mechanisms of pathogenesis or antidrug resistance in fungi, by introducing alleles of different species [29]. Moreover, *H. capsulatum* uses a recombinant heterothallic sexual reproductive system, where its *MAT1* locus participates in a mating type-specific manner [30, 31]. The *MAT1* locus of *H. capsulatum* has two different idiomorphs (MAT1-1 and MAT1-2) that represent each *H. capsulatum* mating type and that are responsible for fungal sexuality. Fungal isolates with MAT1-1 or MAT1-2 mating types coexist in nature, but they are not equally distributed in different geographic areas [32, 33]. Another reason for such high genetic diversity in *Histoplasma* could be the presence of mutations, although these occur with very low frequency under natural conditions. An additional cause that could contribute to the genetic diversity of this fungus is the preponderant role of bats in spreading *H. capsulatum* in different environments, which could favour a genetic exchange between isolates from different sources and geographic origins (either at short or large distances), facilitating fungal gene flow. This idea is compatible with a possible coevolution between the pathogen (*H. capsulatum*) and its wild host (such as bats) [2, 3, 7, 34, 35].

In this study, our results highlight the existence of clonal populations in Latin America, because all Argentinian *H*. *capsulatum* isolates (subgroup 1b) showed low genetic diversity (see Table 2) and the *I*_A_ value obtained for these isolates supports a clonal population structure. This close relationship between the Argentinian *H*. *capsulatum* isolates was previously suggested by Landaburu *et al*. [36], who proposed the possible presence of a clonal population in geographic regions of Argentina, associated with particular microniches.

It is important to mention that our RAPD-PCR results were well-sustained by a value of *CCC*r = 0.999. As we expected, the reference strains from Panama (lineage G-186B) and the USA (NAm 2 (G-217B), NAm 1 (Downs)) did not show a close relationship with the Latin American isolates studied. Regarding isolates GeM from Colombia and EH-319 from Mexico, which did not group according to their geographic origin it is possible that their corresponding patients have acquired histoplasmosis infections at a location different from where they lived. Occasionally, when using *H. capsulatum* clinical isolates, defining the area of a patient's infection original source may provide difficulties due to the erratic migration of humans, which introduces bias into the geographic origin information of the isolates. However, it is also possible that isolates GeM (Colombia) and EH-319 (Mexico) exhibited genetic exchanges (introgression) in different sympatric populations.

In conclusion, this paper widens our knowledge of the distribution of *H*. *capsulatum* populations in Latin America, through the investigation of clinical isolates according to their geographic origin and population structures. In this study, using a statistical analysis (association index), we demonstrated, for the first time, the existence of a clonal population structure in the Argentinian *H. capsulatum* isolates. Furthermore, fungal populations from Mexico and Colombia showed the highest genetic diversity, which is compatible with a recombining population structure, as it was reported elsewhere. Finally, our findings contribute to the molecular epidemiology of histoplasmosis in Latin American countries.
